# 4264 Early Childhood and Prepubertal Overweight and Obesity are Associated with Earlier Pubertal Onset in Boys and Girls: A Prospective Birth Cohort Study

**DOI:** 10.1017/cts.2020.123

**Published:** 2020-07-29

**Authors:** Li-Kuang Chen, Guoying Wang, Wendy Bennett, Xiaobin Wang

**Affiliations:** 1 Johns Hopkins University School of Medicine; 2 Johns Hopkins University, School of Public Health

## Abstract

OBJECTIVES/GOALS: It is hypothesized that the global secular trend toward earlier puberty onset, with implications for many future health outcomes, is related to the obesity epidemic. This study aims to examine prospective associations between weight during specific developmental windows and timing of puberty onset. METHODS/STUDY POPULATION: This study includes 1,296 mother-infant dyads from the Boston Birth Cohort, a predominantly minority (>80% black/Hispanic), low-income, and urban prospective birth cohort recruited and followed between 1998 and 2019. Age at peak height velocity (APHV), a well-defined and standardized proxy for puberty onset, is derived by fitting height measurements recorded during clinical visits using a mixed effects growth curve model. Multiple linear regression is performed to examine the relationships between early childhood (ages 2-5y) and prepubertal (ages 6-9y) overweight and obesity, weight trajectories between these two periods, and APHV, while controlling for known contributors to early puberty. RESULTS/ANTICIPATED RESULTS: Compared to counterparts with normal BMIs, kids who were obese during ages 2-5y (boys: −0.21y, CI[−0.39, −0.04]; girls: −0.22y, CI[−0.39, −0.05]) or ages 6-9y (boys: −0.27y, CI[−0.43, −0.11]; girls: −0.37y, CI[−0.52, −0.23]) had an earlier APHV. Being overweight during ages 6-9y was also associated an earlier APHV (boys: −0.26y, CI[−0.46, −0.07]; girls: −0.26y, CI[−0.42, −0.10]). Looking at weight trajectories, kids who were persistently overweight or obese from ages 2-5y to ages 6-9y had an earlier APHV (boys: −0.28y, CI[−0.45, −0.12]; girls: −0.31y, CI[−0.46, −0.16]), as did girls with normal BMIs during ages 2-5y and who were overweight or obese during ages 6-9y (−0.45y CI[−0.64, −0.26]). DISCUSSION/SIGNIFICANCE OF IMPACT: The temporal and dose-response relationships seen in this historically understudied population suggests that childhood obesity is etiologically important in the development, and even programming, of early puberty. This has implications for prediction, prevention, and mitigation of health disparities.

